# Supplementary material for: miR-129-5p as a biomarker for pathology and cognitive decline in Alzheimer’s disease
Source: Alzheimers Res Ther. 2024 Jan 9;16:5. doi: 10.1186/s13195-023-01366-8 (PMC10775662; doi:10.1186/s13195-023-01366-8)
Supplement: Supplementary file 1 — Additional file 1. [file 13195_2023_1366_MOESM1_ESM.docx]

**Supplemental table 1**. Demographic information of participants from GSE157239 dataset

|  | **NCI**  **(n=8)** | **AD**  **(n=8)** | **Total**  **(n=16)** | ***P* value ^c^** |
| --- | --- | --- | --- | --- |
| **Female ^a^** | 5 (62.5) | 6 (75) | 11 (68.8) | 0.59 |
| **Age at death in years ^b^** | 81.5 (76.0 – 83.8) | 81.5 (75.8 – 84.5) | 81.5 (75.8 – 84.5) | 0.96 |

Values are n (%), unless indicated otherwise.

^a^ Data are presented as number (percent)

^b^ Data are shown as median (interquartile range)

^c^ The Mann-Whitney U test or chi-square test was used to determine the *P* value for comparisons between groups, as appropriate.

Abbreviations: AD, Alzheimer’s dementia; NCI, no cognitive impairment.

**Supplemental table 2**. Association analysis results of miRNAs with diagnosis and clinical and pathological traits. Here we showed only significantly differentially expressed miRNAs in AD.

|  | **AD diagnosis** | | **CERAD** | | **Braak** | | **Global cognition**  **at the last visit** | | **Slope of**  **global cognition** | |
| --- | --- | --- | --- | --- | --- | --- | --- | --- | --- | --- |
| **miRNA** | **Estimate ^a^** | ***P* value ^c^** | **Estimate ^a^** | ***P* value ^c^** | **Estimate ^a^** | ***P* value ^c^** | **beta ^b^** | ***P* value ^c^** | **beta ^b^** | ***P* value ^c^** |
| **miR-100** | 1.837 | 0.032* | 0.902 | 0.202 | 0.498 | 0.900 | -0.602 | 0.007** | -0.286 | 0.582 |
| **miR-1260** | 0.650 | 0.047* | 0.352 | 0.127 | 0.154 | 0.900 | -0.144 | 0.124 | -0.086 | 0.621 |
| **miR-129-3p** | -1.816 | 0.009** | -0.760 | 0.113 | -0.624 | 0.620 | 0.809 | < 0.001*** | 0.383 | 0.006** |
| **miR-129-5p** | -3.771 | < 0.001*** | -1.208 | 0.007** | -1.023 | 0.048* | 1.432 | < 0.001*** | 0.757 | < 0.001*** |
| **miR-132** | -2.787 | < 0.001*** | -0.852 | < 0.001*** | -0.686 | 0.001** | 0.899 | < 0.001*** | 0.334 | < 0.001*** |
| **miR-133b** | -1.581 | 0.002** | -0.369 | 0.526 | -0.403 | 0.840 | 0.581 | <0.001*** | 0.219 | 0.180 |
| **miR-19b** | 1.773 | 0.012* | 0.393 | 0.670 | 0.468 | 0.900 | -0.571 | <0.001*** | -0.246 | 0.464 |
| **miR-29a** | 1.811 | 0.021* | 0.322 | 0.756 | 0.487 | 0.900 | -0.775 | <0.001*** | -0.042 | 0.984 |
| **miR-335** | 1.485 | 0.031* | 0.337 | 0.733 | 0.276 | 0.900 | -0.439 | 0.009** | -0.167 | 0.659 |
| **miR-33a** | 1.192 | 0.032* | 0.274 | 0.736 | 0.342 | 0.900 | -0.012 | 0.969 | -0.086 | 0.894 |
| **miR-410** | -1.118 | 0.031* | -0.406 | 0.492 | -0.081 | 0.950 | 0.318 | 0.016* | 0.203 | 0.180 |
| **miR-433** | -1.590 | 0.015* | -0.355 | 0.690 | -0.305 | 0.900 | 0.461 | 0.005** | 0.286 | 0.180 |
| **miR-504** | -1.942 | 0.032* | -0.622 | 0.492 | -0.701 | 0.774 | 0.492 | 0.016* | 0.270 | 0.542 |
| **miR-519a** | 1.573 | 0.012* | 0.469 | 0.492 | 0.261 | 0.900 | -0.473 | 0.002** | -0.142 | 0.659 |
| **miR-99b** | 1.463 | 0.031* | 0.800 | 0.113 | 0.384 | 0.900 | -0.596 | <0.001*** | -0.314 | 0.148 |

^a^ Logistic regression coefficient of associations between miRNAs and diagnosis and pathological traits

^b^ Linear regression coefficients of association between miRNAs and cognitive traits.

^c^ Adjusted *p* value for FDR

Abbreviations: AD, Alzheimer’s dementia; CERAD, Consortium to Establish a Registry for Alzheimer’s Disease; FDR, false discovery rate; miRNAs, microRNAs.

Note: significance stars indicating the *p* values of the correlations adjustment for multiple comparisons

* *P*-value ≤ 0.05.

** *P*-value ≤ 0.01.

*** *P*-value ≤ 0.001.

**Supplemental table 3**. Association analysis results of module eigengenes with diagnosis and clinical and pathological traits.

|  | **AD diagnosis** | | **CERAD** | | **Braak** | | **Global cognition**  **at the last visit** | | **Slope of**  **global cognition** | |
| --- | --- | --- | --- | --- | --- | --- | --- | --- | --- | --- |
| **Module** | **Estimate ^a^** | ***P* value ^c^** | **Estimate ^a^** | ***P* value ^c^** | **Estimate ^a^** | ***P* value ^c^** | **beta ^b^** | ***P* value ^c^** | **beta ^b^** | ***P* value ^c^** |
| **M0** | 4.794 | 0.303 | 1.101 | 0.818 | 3.236 | 0.271 | -0.542 | 0.851 | -0.280 | 0.870 |
| **M1** | -5.743 | 0.298 | -0.496 | 0.818 | 0.549 | 0.913 | -0.216 | 0.851 | 0.927 | 0.646 |
| **M2** | -0.593 | 0.872 | -1.623 | 0.818 | -0.232 | 0.913 | 1.142 | 0.638 | 0.150 | 0.870 |
| **M3** | -19.480 | 0.005** | -8.393 | 0.034* | -7.458 | 0.067 | 5.100 | 0.008** | 3.830 | 0.017* |

^a^ Logistic regression coefficient of associations between miRNAs and diagnosis and pathological traits

^b^ Linear regression coefficients of association between miRNAs and cognitive traits.

^c^ Adjusted *p* value for FDR

Abbreviations: AD, Alzheimer’s dementia; CERAD, Consortium to Establish a Registry for Alzheimer’s Disease; FDR, false discovery rate; M, module.

Note: significance stars indicating the *p* values of the correlations adjustment for multiple comparisons

* *P*-value ≤ 0.05.

** *P*-value ≤ 0.01.

**Supplemental table 4.** Enrichment analysis of miRNAs in modules from the ROS/MAP dataset

| **Biological process** | **Number of miRNAs**  **belonging to the pathway** | **Fold enrichment** | ***P* value ^a^** |
| --- | --- | --- | --- |
| **M1 module** | | | |
| Innate Immunity | 27 | 7.024 | < 0.001 |
| Skeletal Muscle Cell Differentiation | 16 | 7.946 | < 0.001 |
| Brain Development | 19 | 5.766 | < 0.001 |
| Cell Division | 13 | 8.355 | < 0.001 |
| Latent Virus Replication | 13 | 8.355 | < 0.001 |
| Glucose Metabolism | 16 | 6.243 | < 0.001 |
| Adipocyte Differentiation | 19 | 5.063 | < 0.001 |
| T-helper 17 Cell Differentiation | 13 | 7.476 | < 0.001 |
| Osteoclast Differentiation | 11 | 8.585 | < 0.001 |
| Neuron Apoptosis | 11 | 8.012 | < 0.001 |
| Bone Regeneration | 15 | 5.463 | < 0.001 |
| T-Cell Differentiation | 11 | 7.512 | < 0.001 |
| Lipid Metabolism | 18 | 4.370 | < 0.001 |
| Nephrotoxicity | 11 | 7.070 | < 0.001 |
| Neuron Differentiation | 10 | 7.804 | < 0.001 |
| Cardiac Regeneration | 12 | 5.960 | < 0.001 |
| Adipogenesis | 11 | 6.009 | < 0.001 |
| Muscle Development | 11 | 5.723 | < 0.001 |
| Cell Migration | 8 | 7.946 | < 0.001 |
| Smooth Muscle Cell Proliferation | 10 | 6.070 | < 0.001 |
| Osteoblast Differentiation | 11 | 4.807 | < 0.001 |
| Cell Motility | 10 | 5.203 | < 0.001 |
| Hepatic Stellate Cell Differentiation | 5 | 10.926 | < 0.001 |
| Autophagy | 6 | 8.194 | < 0.001 |
| Chondrocyte Development | 8 | 5.827 | < 0.001 |
| Osteoclastogenesis | 5 | 9.105 | 0.002 |
| Cholesterol Homeostasis | 6 | 7.284 | 0.002 |
| Circadian Rhythm | 9 | 4.470 | 0.003 |
| Regulation of Nf-Κb Pathway | 5 | 7.804 | 0.006 |
| Cellular Senescence | 5 | 7.804 | 0.006 |
| Myogensis | 5 | 6.829 | 0.015 |
| Transdifferentiation | 5 | 6.829 | 0.015 |
| Cell Adhesion | 5 | 6.829 | 0.015 |
| Cholesterol Metabolism | 5 | 6.829 | 0.015 |
| Plasma Cell Differentiation | 5 | 6.829 | 0.015 |
| Embryonic Development | 7 | 4.499 | 0.023 |
| Vascular Smooth Muscle Cell Differentiation | 5 | 6.070 | 0.030 |
| Neutrophil Differentiation | 3 | 10.926 | 0.041 |
| Regulation of Wnt Signaling Pathway | 4 | 7.284 | 0.047 |
| **M2 module** | | | |
| Embryonic Development | 5 | 6.941 | 0.005 |
| Circadian Rhythm | 5 | 5.364 | 0.020 |
| Chondrocyte Development | 4 | 6.293 | 0.031 |
| DNA Damage Response | 4 | 5.900 | 0.040 |
| **M3 module** | | | |
| Muscle Development | 4 | 7.492 | 0.013 |
| Glucose Metabolism | 4 | 5.619 | 0.038 |
| Neuron Apoptosis | 3 | 7.867 | 0.045 |
| Bone Regeneration | 4 | 5.244 | 0.049 |

^a^ Adjusted p value for Bonferroni correction

Abbreviations: M, module; MAP, Memory and Aging Project; miRNAs, microRNAs; ROS, Religious Orders Study.

**Supplemental table 5**. Association analysis results of top 10 candidate hub miRNAs with diagnosis and clinical and pathological traits

|  | **AD diagnosis** | | **CERAD** | | **Braak** | | **Global cognition**  **at the last visit** | | **Slope of**  **global cognition** | |
| --- | --- | --- | --- | --- | --- | --- | --- | --- | --- | --- |
| **Hub miRNAs** | **Estimate ^a^** | ***P* value ^c^** | **Estimate ^a^** | ***P* value ^c^** | **Estimate ^a^** | ***P* value ^c^** | **beta ^b^** | ***P* value ^c^** | **beta ^b^** | ***P* value ^c^** |
| **miR-103** | -0.258 | 0.845 | 0.087 | 0.910 | -0.103 | 0.778 | 0.027 | 0.853 | 0.100 | 0.573 |
| **miR-1260** | 0.650 | 0.008** | 0.352 | 0.012* | 0.154 | 0.450 | -0.144 | 0.035* | -0.086 | 0.297 |
| **miR-200a** | 0.705 | 0.046* | 0.413 | 0.025* | 0.182 | 0.450 | -0.195 | 0.035* | -0.040 | 0.609 |
| **miR-744** | 0.317 | 0.603 | 0.304 | 0.239 | 0.095 | 0.778 | -0.279 | 0.013* | -0.047 | 0.609 |
| **miR-107** | -0.214 | 0.845 | 0.031 | 0.910 | -0.170 | 0.778 | 0.095 | 0.571 | 0.107 | 0.573 |
| **let-7e** | 0.081 | 0.896 | 0.056 | 0.910 | -0.157 | 0.778 | -0.390 | 0.057 | -0.028 | 0.863 |
| **miR-2117** | -0.091 | 0.845 | -0.035 | 0.910 | -0.046 | 0.778 | 0.102 | 0.309 | 0.119 | 0.297 |
| **miR-433** | -1.590 | 0.002** | -0.355 | 0.258 | -0.305 | 0.450 | 0.461 | 0.001** | 0.286 | 0.033* |
| **miR-221** | -1.084 | 0.046* | -0.368 | 0.303 | -0.429 | 0.450 | 0.256 | 0.114 | 0.150 | 0.416 |
| **miR-129-5p** | -3.771 | < 0.001*** | -1.208 | < 0.001*** | -1.023 | 0.003** | 1.432 | < 0.001*** | 0.757 | < 0.001*** |

^a^ Logistic regression coefficient of associations between miRNAs and diagnosis and pathological traits

^b^ Linear regression coefficients of association between miRNAs and cognitive traits.

^c^ Adjusted *p* value for FDR

Abbreviations: AD, Alzheimer’s dementia; CERAD, Consortium to Establish a Registry for Alzheimer’s Disease; FDR, false discovery rate; miRNAs, microRNAs.

Note: significance stars indicating the *p* values of the correlations adjustment for multiple comparisons

* *P*-value ≤ 0.05.

** *P*-value ≤ 0.01.

*** *P*-value ≤ 0.001.

**Supplemental table 6**. Gene ontology analysis of target genes of five AD-associated hub miRNAs (miR-129-5p, miR-1260, miR-200a, miR-433, and miR-221)

| **Biological process** | **Number of target genes belonging to the pathway** | **Fold enrichment** | ***P* value ^a^** |
| --- | --- | --- | --- |
| Regulation of transcription, DNA-templated | 111 | 1.991 | < 0.001 |
| Positive regulation of transcription from RNA polymerase II promoter | 122 | 1.800 | < 0.001 |
| Regulation of transcription from RNA polymerase II promoter | 157 | 1.634 | < 0.001 |
| Negative regulation of transcription from RNA polymerase II promoter | 102 | 1.861 | < 0.001 |
| Regulation of alternative mRNA splicing, via spliceosome | 19 | 5.351 | < 0.001 |
| Protein phosphorylation | 63 | 2.119 | < 0.001 |
| Nervous system development | 53 | 2.286 | < 0.001 |
| Chromatin organization | 39 | 2.475 | < 0.001 |
| Regulation of RNA splicing | 16 | 3.561 | 0.010 |
| Neuron migration | 20 | 2.955 | 0.012 |
| Positive regulation of mitotic cell cycle | 10 | 5.462 | 0.016 |

^a^ Adjusted p value for Bonferroni correction

Abbreviations: AD, Alzheimer’s dementia; miRNAs, microRNAs; mRNA, messenger RNAs.

**Supplemental table 7**. kMEs of all miRNAs for the CM2 module in the replication (GEO) dataset

| **miRNA** | **kME** | ***P* value ^a^** |
| --- | --- | --- |
| **miR-744** | 0.946 | 1.02 × 10^-6^ |
| **let-7e** | 0.892 | 4.60 × 10^-5^ |
| **miR-485-3p** | 0.887 | 4.60 × 10^-5^ |
| **miR-769-3p** | 0.883 | 4.60 × 10^-5^ |
| **miR-491-5p** | 0.881 | 4.60 × 10^-5^ |
| **miR-331-3p** | 0.863 | 9.46 × 10^-5^ |
| **miR-107** | 0.850 | 1.51 × 10^-4^ |
| **miR-129-5p** | 0.833 | 2.58 × 10^-4^ |
| **miR-221** | 0.827 | 2.77 × 10^-4^ |
| **miR-129-3p** | 0.825 | 2.77 × 10^-4^ |
| **miR-770-5p** | 0.823 | 2.77 × 10^-4^ |
| **miR-708** | 0.807 | 4.55 × 10^-4^ |
| **miR-487a** | 0.753 | 1.99 × 10^-3^ |
| **miR-125a-5p** | 0.716 | 4.42 × 10^-3^ |
| **miR-132** | 0.692 | 6.71 × 10^-3^ |
| **miR-103** | 0.684 | 7.36 × 10^-3^ |
| **miR-31** | 0.637 | 1.59 × 10^-2^ |
| **miR-34a** | 0.629 | 1.71 × 10^-2^ |
| **miR-99b** | 0.608 | 2.24 × 10^-2^ |
| **miR-1275** | 0.587 | 2.61 × 10^-2^ |
| **miR-140-5p** | 0.558 | 3.65 × 10^-2^ |
| **miR-543** | 0.529 | 4.97 × 10^-2^ |
| **miR-767-5p** | 0.497 | 6.82 × 10^-2^ |
| **miR-379** | 0.467 | 7.97 × 10^-2^ |
| **miR-186** | 0.454 | 8.69 × 10^-2^ |
| **miR-548a-3p** | 0.450 | 8.69 × 10^-2^ |
| **miR-525-5p** | 0.448 | 8.69 × 10^-2^ |
| **miR-518e** | 0.398 | 1.30 × 10^-1^ |
| **miR-603** | 0.391 | 1.34 × 10^-1^ |
| **miR-548h** | -0.474 | 7.73 × 10^-2^ |
| **miR-370** | -0.485 | 7.20 × 10^-2^ |
| **miR-200a** | -0.492 | 6.93 × 10^-2^ |
| **miR-1246** | -0.592 | 2.53 × 10^-2^ |
| **miR-518f** | -0.602 | 2.30 × 10^-2^ |

^a^ Adjusted *p* value using FDR

kME and *p* values represent the correlation and significance levels, respectively, between the miRNA expression levels and module eigengene of the CM2 module.

Abbreviations: CM, consensus module; FDR, false discovery rate; GEO, Gene Expression Omnibus; ME, module eigengene; miRNAs, microRNAs.

**Supplemental table 8**. kMEs of all miRNAs for the CM3 module in the replication (GEO) dataset

| **miRNA** | **kME** | ***P* value ^a^** |
| --- | --- | --- |
| **miR-346** | 0.951 | 2.25 × 10^-7^ |
| **miR-139-5p** | 0.932 | 2.41 × 10^-6^ |
| **miR-191** | 0.900 | 2.21 × 10^-5^ |
| **miR-423-3p** | 0.878 | 6.29 × 10^-5^ |
| **miR-361-5p** | 0.854 | 1.63 × 10^-4^ |
| **miR-127-3p** | 0.851 | 1.63 × 10^-4^ |
| **miR-92b** | 0.843 | 1.98 × 10^-4^ |
| **miR-30c** | 0.803 | 6.65 × 10^-4^ |
| **miR-885-5p** | 0.800 | 6.65 × 10^-4^ |
| **miR-423-5p** | 0.800 | 6.65 × 10^-4^ |
| **miR-138** | 0.786 | 8.90 × 10^-4^ |
| **miR-149** | 0.784 | 8.90 × 10^-4^ |
| **miR-23b** | 0.766 | 1.39 × 10^-3^ |
| **miR-324-3p** | 0.751 | 1.90 × 10^-3^ |
| **miR-197** | 0.726 | 3.17 × 10^-3^ |
| **miR-487b** | 0.692 | 5.50 × 10^-3^ |
| **miR-128** | 0.689 | 5.50 × 10^-3^ |
| **miR-30d** | 0.689 | 5.50 × 10^-3^ |
| **miR-93** | 0.666 | 8.07 × 10^-3^ |
| **miR-520d-3p** | 0.656 | 9.13 × 10^-3^ |
| **miR-1286** | 0.651 | 9.43 × 10^-3^ |
| **miR-574-3p** | 0.632 | 1.23 × 10^-2^ |
| **miR-124** | 0.624 | 1.28 × 10^-2^ |
| **miR-185** | 0.624 | 1.28 × 10^-2^ |
| **miR-425** | 0.607 | 1.62 × 10^-2^ |
| **miR-329** | 0.585 | 2.12 × 10^-2^ |
| **miR-22** | 0.539 | 3.59 × 10^-2^ |
| **miR-125a-3p** | 0.538 | 3.59 × 10^-2^ |
| **miR-484** | 0.525 | 4.05 × 10^-2^ |
| **miR-154** | 0.381 | 1.49 × 10^-1^ |
| **miR-142-3p** | -0.371 | 1.57 × 10^-1^ |
| **miR-190b** | -0.427 | 1.05 × 10^-1^ |
| **miR-224** | -0.719 | 3.49 × 10^-3^ |

^a^ Adjusted *p* value using FDR

kME and *p* values represent the correlation and significance levels, respectively, between the miRNA expression levels and module eigengene of the CM3 module.

Abbreviations: CM, consensus module; FDR, false discovery rate; GEO, Gene Expression Omnibus; ME, module eigengene; miRNAs, microRNAs.

**Supplemental table 9.** Enrichment analysis of miRNAs in consensus modules from the combined ROS/MAP and GEO datasets

| **Biological process** | **Number of miRNAs**  **belonging to the pathway** | **Fold enrichment** | ***P* value ^a^** |
| --- | --- | --- | --- |
| **CM1 module** | | | |
| Innate Immunity | 23 | 14.048 | < 0.001 |
| Adipocyte Differentiation | 17 | 10.636 | < 0.001 |
| Latent Virus Replication | 11 | 16.598 | < 0.001 |
| T-Cell Differentiation | 10 | 16.033 | < 0.001 |
| Chondrocyte Development | 9 | 15.391 | < 0.001 |
| Cell Motility | 10 | 12.215 | < 0.001 |
| Cell Division | 9 | 13.581 | < 0.001 |
| Adipogenesis | 9 | 11.543 | < 0.001 |
| Glucose Metabolism | 10 | 9.161 | < 0.001 |
| Circadian Rhythm | 9 | 10.494 | < 0.001 |
| Embryonic Stem Cell Differentiation | 10 | 8.275 | < 0.001 |
| Osteoclastogenesis | 5 | 21.377 | < 0.001 |
| Muscle Development | 8 | 9.772 | < 0.001 |
| Cardiac Regeneration | 8 | 9.328 | < 0.001 |
| Bone Regeneration | 9 | 7.696 | < 0.001 |
| Regulation of Nf-Κb Pathway | 5 | 18.323 | < 0.001 |
| Osteoblast Differentiation | 8 | 8.209 | < 0.001 |
| Smooth Muscle Cell Proliferation | 7 | 9.976 | < 0.001 |
| T-helper 17 Cell Differentiation | 7 | 9.451 | < 0.001 |
| Vascular Smooth Muscle Cell Differentiation | 5 | 14.251 | < 0.001 |
| Neuron Apoptosis | 6 | 10.261 | < 0.001 |
| Skeletal Muscle Cell Differentiation | 7 | 8.161 | < 0.001 |
| DNA Damage Response | 6 | 9.620 | < 0.001 |
| Nephrotoxicity | 6 | 9.054 | < 0.001 |
| Smooth Muscle Cell Differentiation | 3 | 25.652 | 0.002 |
| Neutrophil Differentiation | 3 | 25.652 | 0.002 |
| Plasma Cell Differentiation | 4 | 12.826 | 0.005 |
| Autophagy | 4 | 12.826 | 0.005 |
| Ovarian Follicle Development | 3 | 19.239 | 0.008 |
| Lipid Metabolism | 8 | 4.560 | 0.009 |
| Brain Development | 7 | 4.988 | 0.013 |
| Hepatic Stellate Cell Differentiation | 3 | 15.391 | 0.020 |
| DNA Damage Repair | 5 | 6.751 | 0.023 |
| **CM2 module** | | | |
| Glucose Metabolism | 5 | 8.104 | 0.001 |
| Cell Migration | 3 | 12.378 | 0.006 |
| Lipid Metabolism | 5 | 5.043 | 0.010 |
| **CM3 module** | | | |
| Regulation of Wnt Signaling Pathway | 4 | 31.053 | < 0.001 |
| Transdifferentiation | 4 | 23.289 | < 0.001 |
| Cholesterol Homeostasis | 4 | 20.702 | < 0.001 |
| Smooth Muscle Cell Proliferation | 5 | 12.939 | < 0.001 |
| Brain Development | 6 | 7.763 | 0.001 |
| Osteoclast Differentiation | 4 | 13.308 | 0.002 |
| Neuron Apoptosis | 4 | 12.421 | 0.003 |
| Glucose Metabolism | 5 | 8.318 | 0.004 |
| Embryonic Development | 4 | 10.960 | 0.006 |
| Neuron Differentiation | 3 | 9.981 | 0.045 |

^a^ Adjusted p value for Bonferroni correction

Abbreviations: CM, consensus module; GEO, Gene Expression Omnibus; MAP, Memory and Aging Project; miRNAs, microRNAs; ROS, Religious Orders Study.

**Supplemental figure 1**. Construction of co-expression network using weighted gene co-expression network analysis


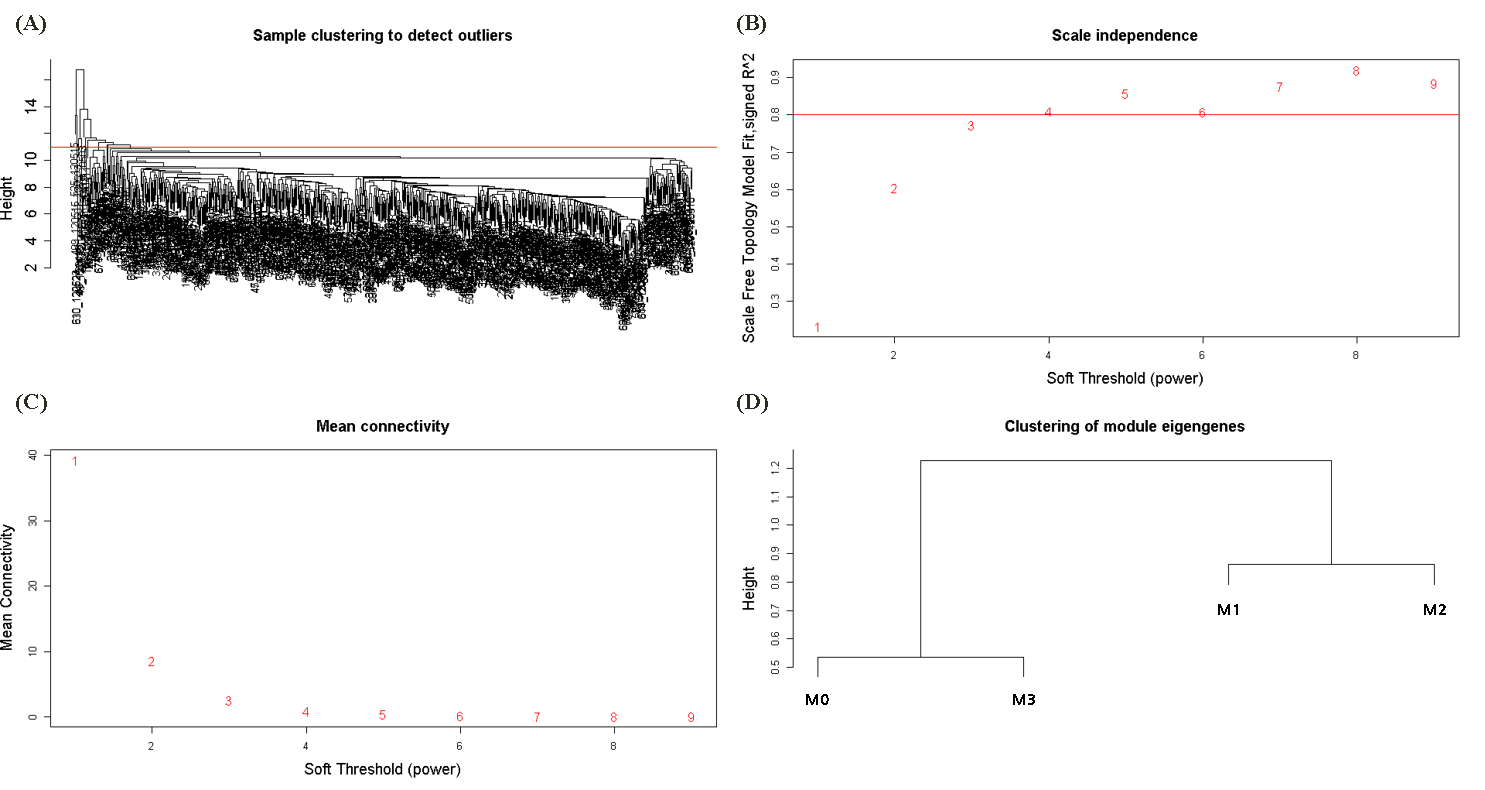


(A) Hierarchical clustering dendrogram of samples in the ROS/MAP dataset. Red line in the dendrogram represents the cutting height to detect outliers. A cutting tree height of 11 was selected, and the number of samples under the red line after clustering was 663. (B) The plot showing the scale-free topology fitting index (y-axis) for different soft-thresholding powers (β) (x-axis). (C) Analysis of mean connectivity (degree, y-axis) for various soft-thresholding powers (x-axis). The soft thresholding power of 4 was chosen to best approximate a scale-free topology because the scale-free topology index reached 0.8 and mean connectivity decreased considerably. (D) Eigengene dendrogram. The dynamic tree cut method was used to merge modules with dissimilarity < 25% when the minimum number of miRNAs in modules was set to 30. The M0 and M3 modules are highly related, while the M1 and M2 modules are also related.

Abbreviations: MAP, Memory and Aging Project; ROS, Religious Orders Study.

**Supplemental figure 2**. Co-expression network construction of consensus modules


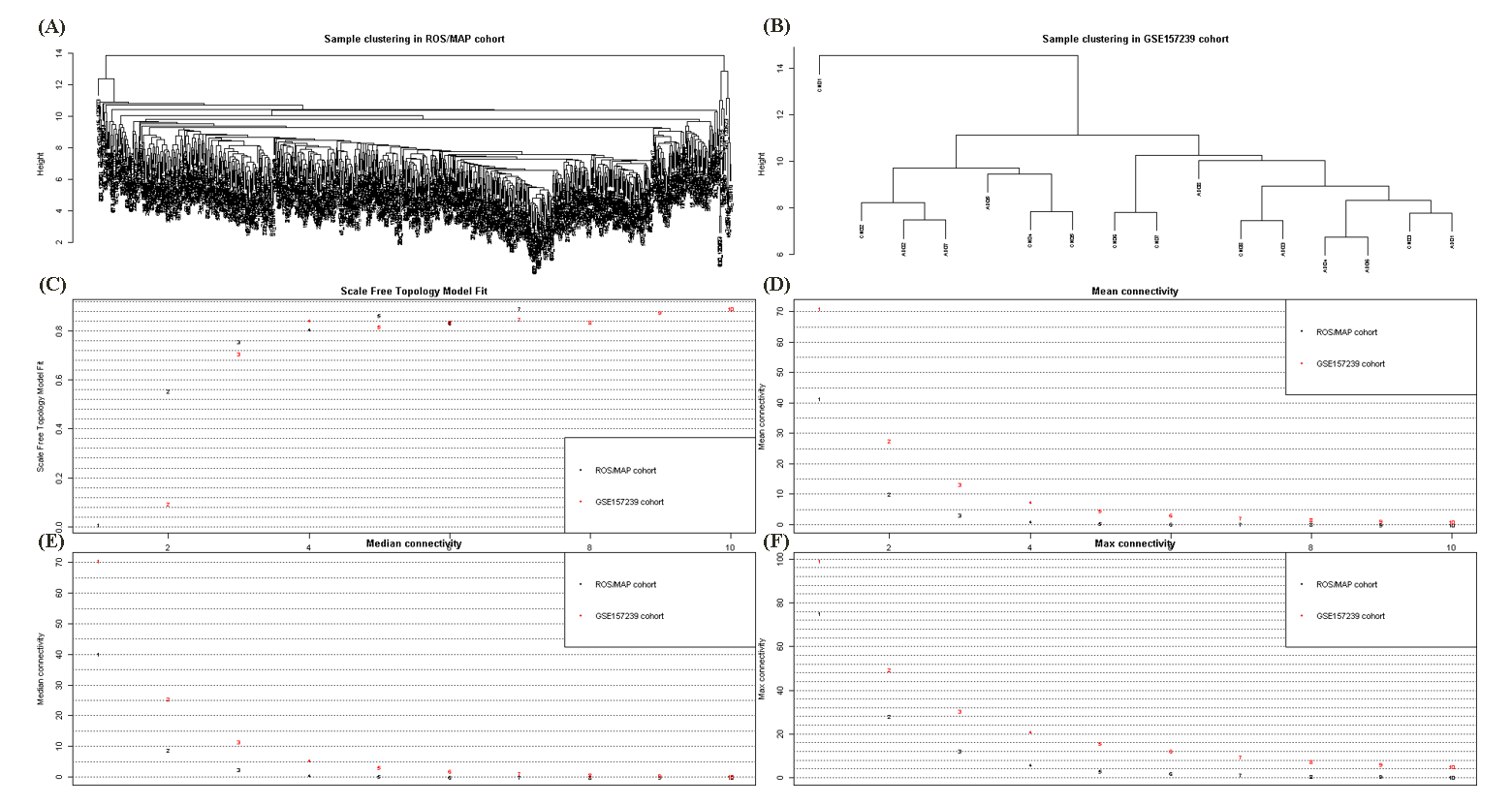


(A, B) Hierarchical clustering dendrogram of samples in the ROS/MAP (A) and GSE157239 (B) datasets. (C) The plot showing the scale-free topology fitting index (y-axis) for different soft-thresholding powers (β) (x-axis). (D to F) Analysis of median, mean, and max connectivity (degree, y-axis) for various soft-thresholding powers (x-axis). The soft thresholding power value of β (= 4) was selected for each dataset to best approximate a scale-free topology because the scale-free topology index reached 0.8 and connectivity measurements decreased considerably.

Abbreviations: MAP, Memory and Aging Project; ROS, Religious Orders Study.

**Supplemental figure 3**. Correspondence of ROS/MAP set-specific and ROS/MAP-GSE157239 consensus modules


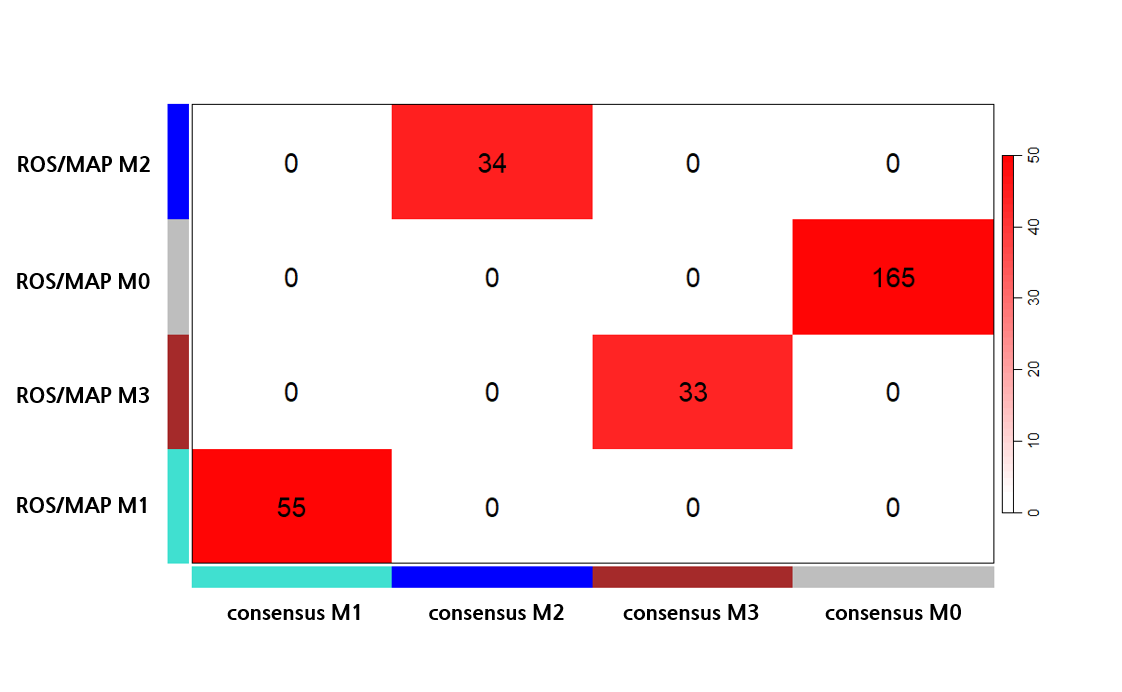


Each row in the table corresponds to a module from the ROS/MAP dataset, and each column to a ROS/MAP-GSE157239 consensus module. The number in each cell represents the count of miRNAs in the corresponding row and column. Color scale indicates the significance of the overlap, with red indicating more significant overlap based on the −log10 transformed *P* values from Fisher’s exact test.

Abbreviations: CM, consensus module; M, module; MAP, Memory and Aging Project; ROS, Religious Orders Study.

**Supplemental figure 4**. Pearson correlation matrix of modules from the ROS/MAP and consensus modules from the combined ROS/MAP and GEO datasets


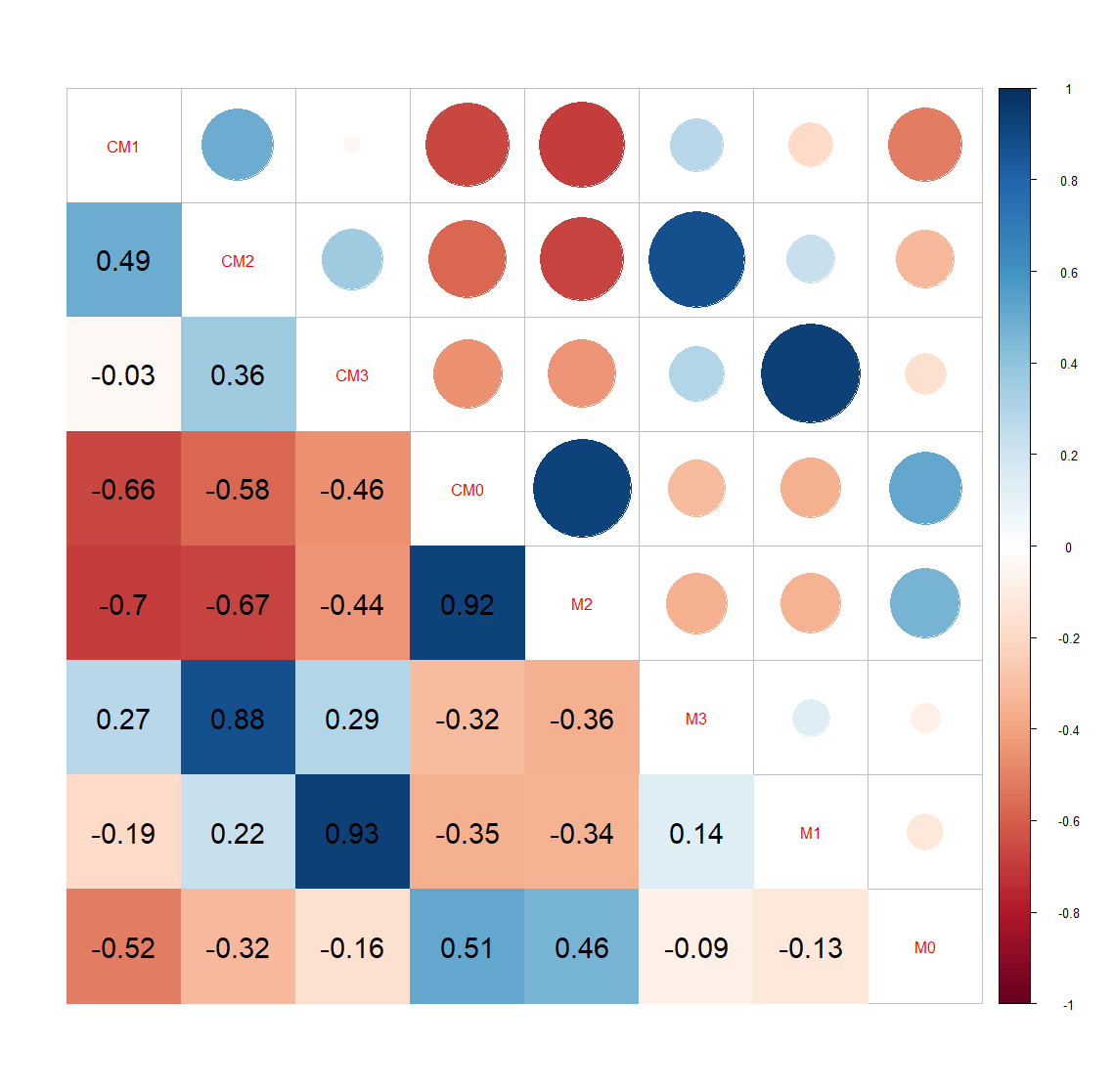


The color scale depicts the strength of the Pearson correlation coefficient. Blue colors represent positive correlations, while red colors denote negative correlations. The darker the color, the stronger the association. Superimposed text reflects the actual numerical value of the Pearson correlation coefficient.

Abbreviations: GEO, Gene Expression Omnibus; MAP, Memory and Aging Project; ROS, Religious Orders Study.
